# Lipoid Proteinosis Masquerading as Seborrheic Dermatitis

**DOI:** 10.7759/cureus.15617

**Published:** 2021-06-13

**Authors:** Alka Tripathi, Sunil Kumar Gupta

**Affiliations:** 1 Department of Ophthalmology, All India Institute of Medical Sciences, Gorakhpur, IND; 2 Department of Dermatology, All India Institute of Medical Sciences, Gorakhpur, IND

**Keywords:** lipoid proteinosis (lp), moniliform blepharosis, seborrheic dermatitis, pathognomonic, ecm1 gene

## Abstract

We report a case of lipoid proteinosis (LP) masquerading as seborrheic dermatitis. A 35-year-old female presented to our outpatient department with complaints of itching and crust-like formation on eyelids for five years. She was treated as a case of seborrheic dermatitis elsewhere and got intermittent relief in itching with medications. Beaded lesions were found along the upper and lower eyelids involving the lash line and caruncle on removing the crust. The verrucous lesions were pathognomonic, moniliform blepharosis of LP. Systemic examination revealed hoarseness of voice, and hyperkeratosis was seen on the dorsum of both of her hands. She has been advised lid hygiene, artificial tears, and antihistaminics for itching. Skin emollients were also advised by dermatologists to decrease the chances of abrasion and bleeding from minor trauma. She was well explained about the danger signs as well as neurological and psychiatric implications of the disease. Although ophthalmologists have a rare encounter with this disease, LP is a well-known entity to dermatologists and otorhinolaryngologists, and thus it may sometimes go undiagnosed. An ophthalmologist should be well aware of the life-threatening complications associated with LP, and patients should be sensitized regarding the chronic nature, supportive measures, and danger signs of the disease.

## Introduction

Lipoid proteinosis (LP) is known as Urbach-Wiethe syndrome or hyalinosis cutis et mucosae and was first reported by dermatologist and otolaryngologist duo Urbach and Wiethe in 1929 [1]. LP is a rare entity with autosomal recessive inheritance and multiple system involvement causing dermatological, otorhinolaryngological, ocular, and neurological manifestations. Presenting symptoms of LP are usually hoarseness of voice due to deposits in the vocal cords [2]. Respiratory insufficiency and upper respiratory tract infections are the sequelae of involvement of the upper aerodigestive tract. Ophthalmological manifestations are pathognomonic of LP, and these are characteristically beaded papules along upper and lower eyelids over the lash line also known as moniliform blepharosis [3]. Skin depositions cause the skin to become thickened and yellowish. Friction areas such as the hands, elbows, knees, buttocks, and armpits bear more brunt, and there is marked hyperkeratosis in response to minor trauma [4]. Although ophthalmologists have a rare encounter with this disease, LP is a pretty well-known entity to dermatologists and otorhinolaryngologists, and thus it may sometimes go undiagnosed. LP often follows a stable, chronic course, and treatment is seldom indicated. The disease although is not life-threatening, but it deteriorates the quality of patients' life. So ophthalmologists should be aware of varied presentation and multidisciplinary approaches for this rare entity [5].

We describe a case of LP masquerading as seborrheic dermatitis and involving bilateral eyelids in a 35-year-old female who presented with the chief complaints of itching and crust-like formation on both eyelids for five years.

## Case presentation

**Figure 1:**
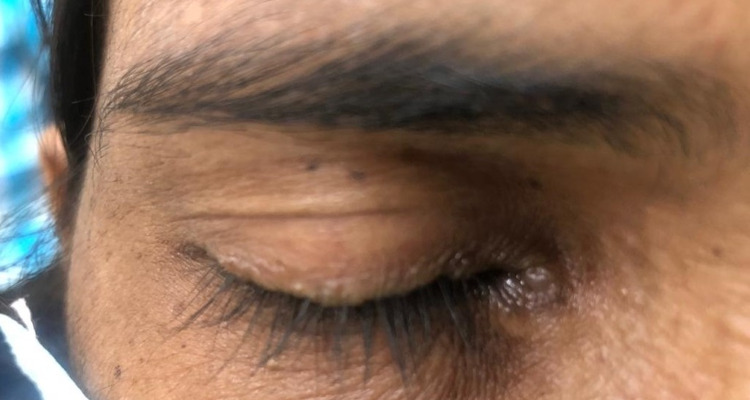
R/E: Beaded lesions were found along the upper and lower eyelids involving the lash line and caruncle R/E, Right eye.

**Figure 2:**
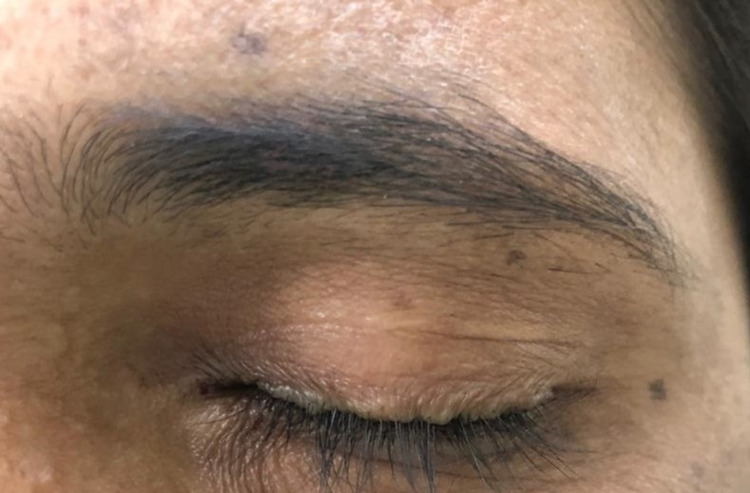
L/E: Beaded lesions were found along the upper and lower eyelids involving the lash line and caruncle L/E, Left eye.

A 35-year-old female presented to our outpatient department with complaints of itching and crust-like formation on eyelids for five years. She was treated as a case of seborrheic dermatitis elsewhere and got intermittent relief in itching with medications. On ophthalmic examination, BCVA (best-corrected visual acuity) was 20/20 in both eyes. Detailed examination revealed dandruff-like material along with the upper and lower eyelid. Beaded lesions were found along the upper and lower eyelids involving the lash line and caruncle on removing the crust. The verrucous lesions were pathognomonic, moniliform blepharosis of LP (Figures 1, 2).

**Figure 3:**
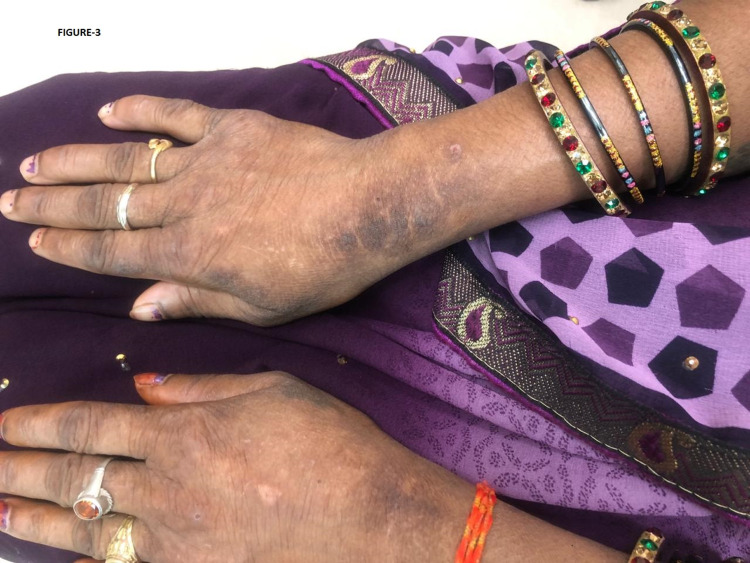
Hyperkeratosis on the dorsum of both of her hands

The lesions were continuous and involved the entire lid margin and caruncle. Intraocular pressure (IOP) and fundus examination were within normal limits. Systemic examination revealed hoarseness of voice, and hyperkeratosis was seen on the dorsum of both of her hands (Figure 3).

For hoarseness of voice, she was sent for an otorhynolaryngologist's opinion, and laryngoscopy revealed thickening and irregularities of the vocal cords' mucosa. She has been advised lid hygiene, artificial tears, and antihistaminics for itching. She has been further counseled regarding the chronic nature of the disease, varied ocular manifestations, and multisystem involvement. Skin emollients were also advised by dermatologists to decrease the chances of abrasion and bleeding from minor trauma. The danger signs as well as neurological and psychiatric implications of the disease were well explained to her.

## Discussion

LP is a rare entity with less than 500 cases being reported in the scientific literature. LP has been reported more from certain areas including Turkey, Iran, and South Africa. The pathogenesis behind this disorder is a mutation of the extracellular-matrix protein-1 (ECM1) gene located on chromosome 1q21 [6]. Faulty ECM1 protein causes reduced binding between ECM1 and other proteins, leading to an unstable extracellular matrix and in turn stimulating neighboring cells to overproduce proteins and other materials. Overproduction of protein leads to their deposition in tissues, which is a characteristic of LP. It is histologically proven that there are intercellular deposits of periodic acid-Schiff (PAS)-positive hyaline material in the skin, mucous membranes, and internal organs.

The hoarseness of voice is usually the presenting symptom in most cases, and it may persist throughout life and in due course of time can cause difficulty or loss of speech. The tongue may be thick and shortened due to deposits. The thickening of the frenulum of the tongue leads to difficulty in extending the tongue. A decrease in taste buds leads to smoothening of the tongue in some cases.

Neurologic manifestations are due to the presence of deposits in the temporal lobe. The most common presentation is recurrent seizures (epilepsy) followed by headaches, agitated behaviors, paranoia, hallucinations, and memory loss.

The lesion's moniliform blepharosis, a pathognomonic feature of LP, can cause irritation or itching of the eyes, but the vision is not hampered in most cases [3]. An ophthalmologist should know to differentiate between moniliform blepharosis and other eyelid disorders due to deposition as their management and underlying systemic condition are quite varied [7-8] (Table 1).

**Table 1:** Different deposition disorders associated with eyelid, common area of involvement, and treatment

Eyelid disorder	Site and presentation	Treatment
Xanthelasma	Lipid-rich deposits along the corners of the eye, usually inner canthus	Lifestyle modification, lasers, radiofrequency ablation
Moniliform blepharosis	Protein deposits along upper and lower eyelids along the lash line	Reassurance, surgical removal, CO_2_ laser therapy
Subepidermal calcinosis	Calcium deposits in the dermis on the nasal aspect of eyelids	Reassurance, surgical removal, CO_2_ laser therapy
Amyloidosis of the eyelid	Waxy yellow or red lesions at corners, which bleed on minor trauma	Biopsy (to rule out malignancy), lubricants, steroids (to control inflammation)

Other ocular manifestations of LP involve the cornea, conjunctiva, sclera, trabecular meshwork, iris/pupil, lens and zonular fibers, retina, and nasolacrimal duct. Infiltration of glands of Zeiss, Moll, and Meibomian by deposits can subsequentially cause madarosis, trichiasis, and sometimes distichiasis. Focal degeneration of macula and drusen formation in Bruch's membrane were observed in about 30%-50% of patients. Glaucoma (deposition of glycoproteins in a trabecular meshwork or due to hyalinization of scleral trabecula), lens dislocation or subluxation, corneal ulceration caused by trichiasis, keratoconus [9], unilateral or bilateral uveitis [10], dry eyes, nasolacrimal duct obstruction, and transient blindness are the uncommon ocular manifestation of LP.

Due to the chronic, progressive, and indolent nature of the disease, no effective treatment is known. In most cases, management is planned according to the site of involvement and presentation. Treatment goals are symptomatic improvement in clinical condition. As in our case, itching was relieved with artificial tears, lid hygiene, and antihistaminic eye drops. Surgical excision of the papules and CO_2_ laser therapy are required mostly for cosmetic purposes.

## Conclusions

LP disease requires a multidisciplinary approach, and consolidated opinions from ophthalmologists, dentists, dermatologists, otorhinolaryngologists, and neurologists are pivotal to lay a firm diagnosis. An ophthalmologist should be well aware of the life-threatening complications associated with LP, and patients should be sensitized regarding the chronic nature, supportive measures, and danger signs of the disease.
